# Renal clear cell carcinoma with co-existing tumor thrombosis of renal vein and ureter: a case report and review of the literature

**DOI:** 10.3389/fonc.2026.1844930

**Published:** 2026-05-13

**Authors:** Qingxuan Li, Shuping Sun, Yanchen Wang, Yuxuan Liu, Yaofei Sun

**Affiliations:** 1 School of Clinical Medicine, Shandong Second Medical University, Weifang, China; 2 Oncology Department, Laixi People’s Hospital, Qingdao, China; 3 Department of Urology, Weifang People’s Hospital, Weifang, Shandong, China

**Keywords:** radical nephrectomy for renal cancer, renal cancer, renal clear cell carcinoma, renal vein tumor thrombus, ureteral tumor thrombus

## Abstract

When renal cell carcinoma locally spreads, it usually progresses along the renal vein and inferior vena cava. However, the spread along the ureteral lumen to the bladder is a rare situation. The author has only collected less than 30 similar cases. There is no standard treatment plan for patients with ureteral metastasis at present, and its pathological significance is also unclear. Here, we describe a 56-year-old male patient who presented to our hospital with fever and gross hematuria. After abdominal computed tomography (CT), we diagnosed him with a left renal malignant tumor and thickening of the left renal pelvis and ureter, considering metastasis. Later, cystoscopy was performed, and a ureteral tumor thrombus protruding into the bladder was found under the microscope. Then, laparoscopic radical nephrectomy of the left kidney and transurethral resection of bladder tumor were performed. After the operation, the patient’s body temperature significantly decreased, and gross hematuria also improved. The pathology and related immunohistochemistry reported clear cell renal cell carcinoma. The patient has been discharged, and we plan to treat him with axitinib and toripalimab combination therapy as the subsequent treatment plan. This case demonstrates that surgical resection is still an effective treatment option for such cases, and we review the relevant literature and discuss the selection of surgical methods and pathological significance.

## Introduction

Renal cell carcinoma (RCC) accounts for less than 3% of all adult malignancies and approximately 85% of malignant renal tumors (1, 2).Most renal cell carcinomas are most likely to metastasize to the bones and lungs, followed by regional lymph nodes, liver, adrenal glands, brain, gallbladder, pancreas and breast (3),Only a very small number of cases will progress along the renal pelvis and ureter to the bladder or form ureteral tumor thrombus. The few cases that invade the bladder are mostly through lymphatic or hematogenous metastasis, and mostly occur several years later (4, 5).At present, it is believed that there are several mechanisms for bladder metastasis of renal cell carcinoma: 1. Direct downward extension along the ureteral lumen; 2. Reverse infiltration along the venous system; 3. Lymphatic spread (6).We describe a case of a male patient whose left renal cell carcinoma directly extended along the ureteral lumen from the renal collecting system to the bladder and formed a ureteral tumor thrombus.

## Case introduction

**Figure 1:**
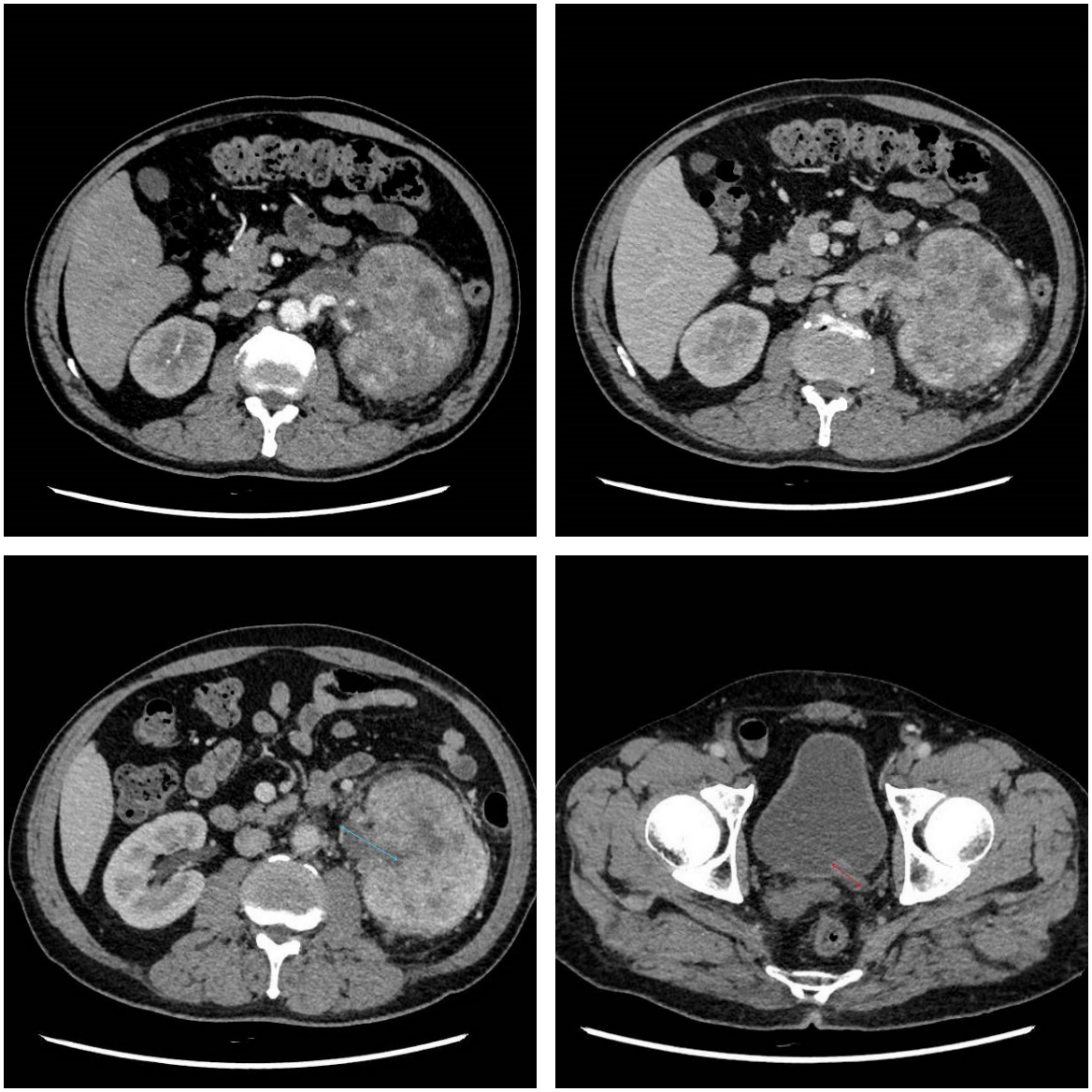
The blue arrow indicates the tumor thrombus within the renal pelvis and proximal ureter. The red arrow shows the ureteral tumor thrombus under CT.

**Figure 2:**
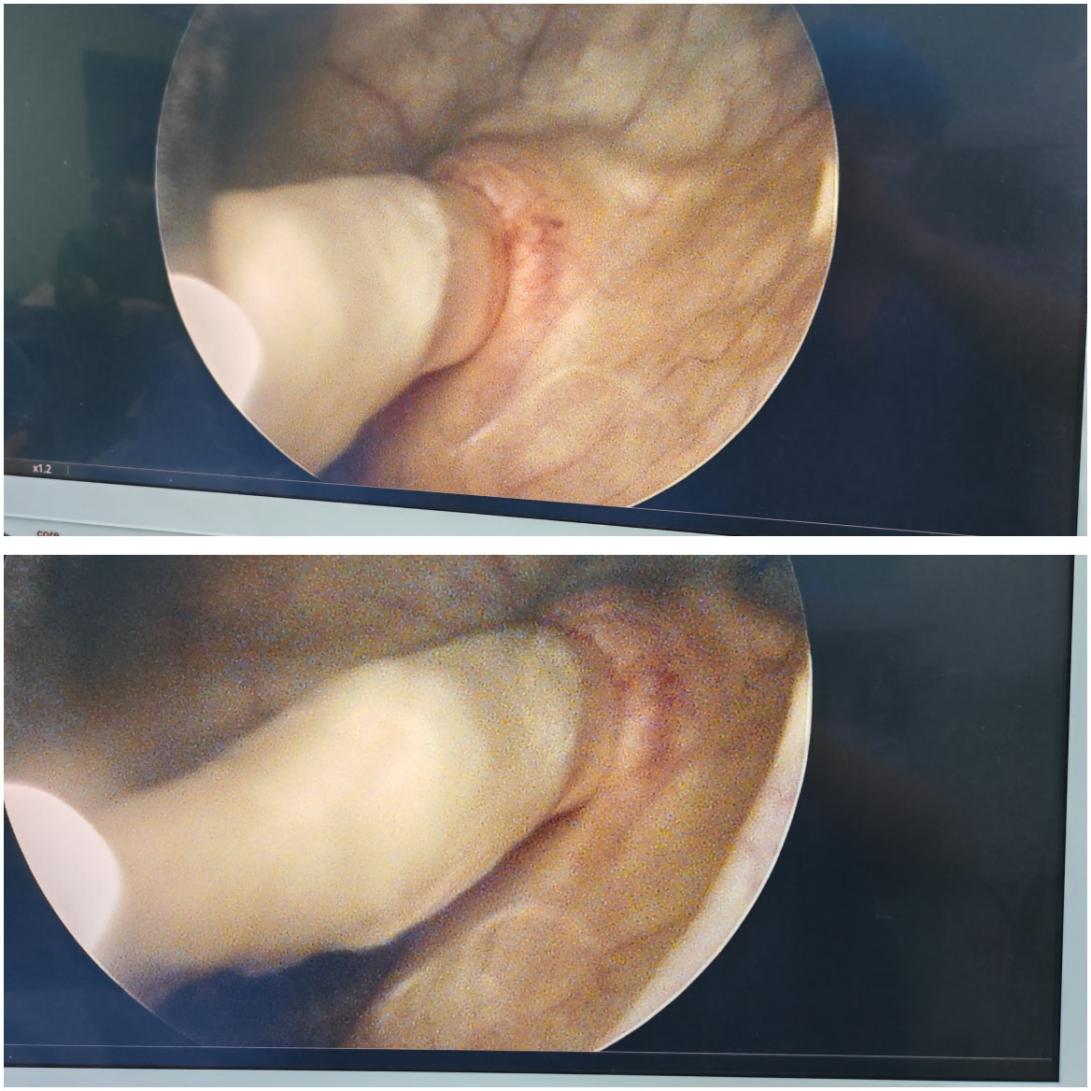
Ureteral tumor thrombus protruding into the bladder under cystoscopy.

A 56-year-old male patient presented to our hospital on January 19, 2026, with persistent fever and gross hematuria. Blood tests including a complete blood count showed an elevated number of monocytes in the white blood cells (10%, 1.2×10^9^/L). Abdominal CT scans, both plain and enhanced, revealed a large tumor in the left kidney with tumor thrombus in the renal vein and ureter, thickening of the tumor thrombus in the left renal vein and left renal pelvis and ureter, and a slightly high-density shadow at the left ureteral orifice; the retroperitoneal lymph nodes were slightly enlarged (**Figure 1**). The patient was admitted to the urology department on the same day. A chest CT scan was performed on the same day and showed multiple lung metastases, with the largest metastatic lesion measuring approximately 1.2 cm in diameter. After discussion by the department head, a cystoscopy was performed on January 22, 2026, which revealed a tumor thrombus in the left ureter extending into the bladder (**Figure 2**). Based on the patient’s condition, the clinical pathological stage was assessed as cT3NxM1. It was decided to first perform surgical resection of the primary lesion for cytoreductive treatment, followed by targeted immunotherapy in subsequent follow-ups.

**Figure 3:**
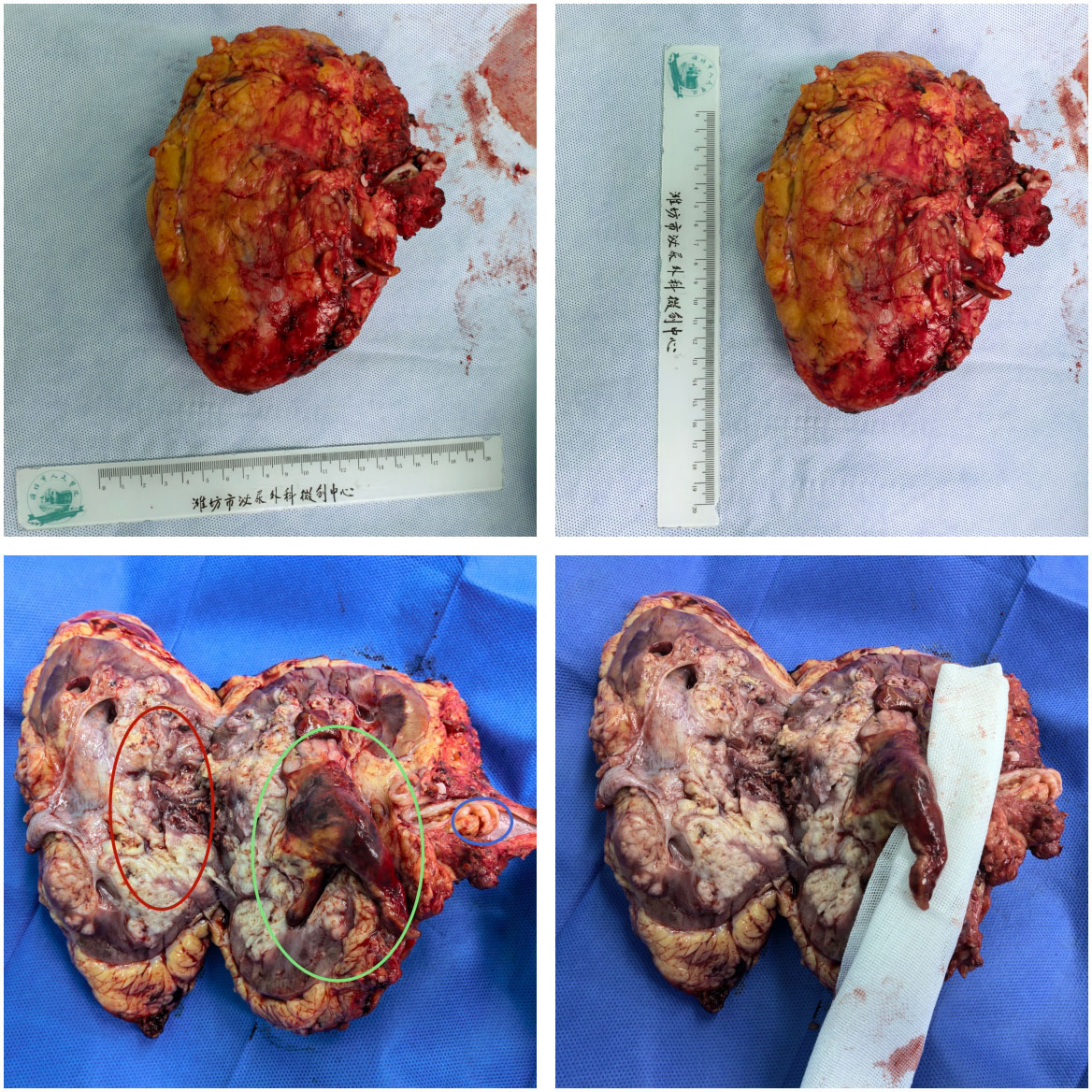
The red circle is the tumor itself. The green circle was ureteral tumor thrombus. The blue circle is the venous tumor thrombus.

Before the operation, the patient had a persistent fever of around 38°C, which was considered as tumor fever. After the preoperative examination was completed and no other contraindications were found, the patient underwent laparoscopic radical resection of left renal carcinoma under general anesthesia with tracheal intubation and cystoscopic bladder tumor resection on January 26, 2026 (**Figure 3**). We first dissociated and severed the left renal artery via the flank approach. Subsequently, a transperitoneal approach was adopted. The left renal hilum was mobilized and divided. The ureter was then mobilized, clamped at its proximal end, and divided, along with division of the left testicular vein. Finally, the entire left renal fat capsule was dissected off en bloc along with the left adrenal gland. However, due to dense adhesions around the distal ureter making dissection difficult, and the presence of distant pulmonary metastasis, the primary objective of this procedure was debulking surgery to remove the primary lesion. Consequently, the distal ureter could not be effectively resected. The remainder of the surgery proceeded smoothly, and the postoperative recovery was uneventful. The patient’s body temperature returned to 36.3°C on the morning of postoperative day 1 and remained at 36.3°Con the morning of postoperative day 2, indicating a prompt resolution of fever.

**Figure 4:**
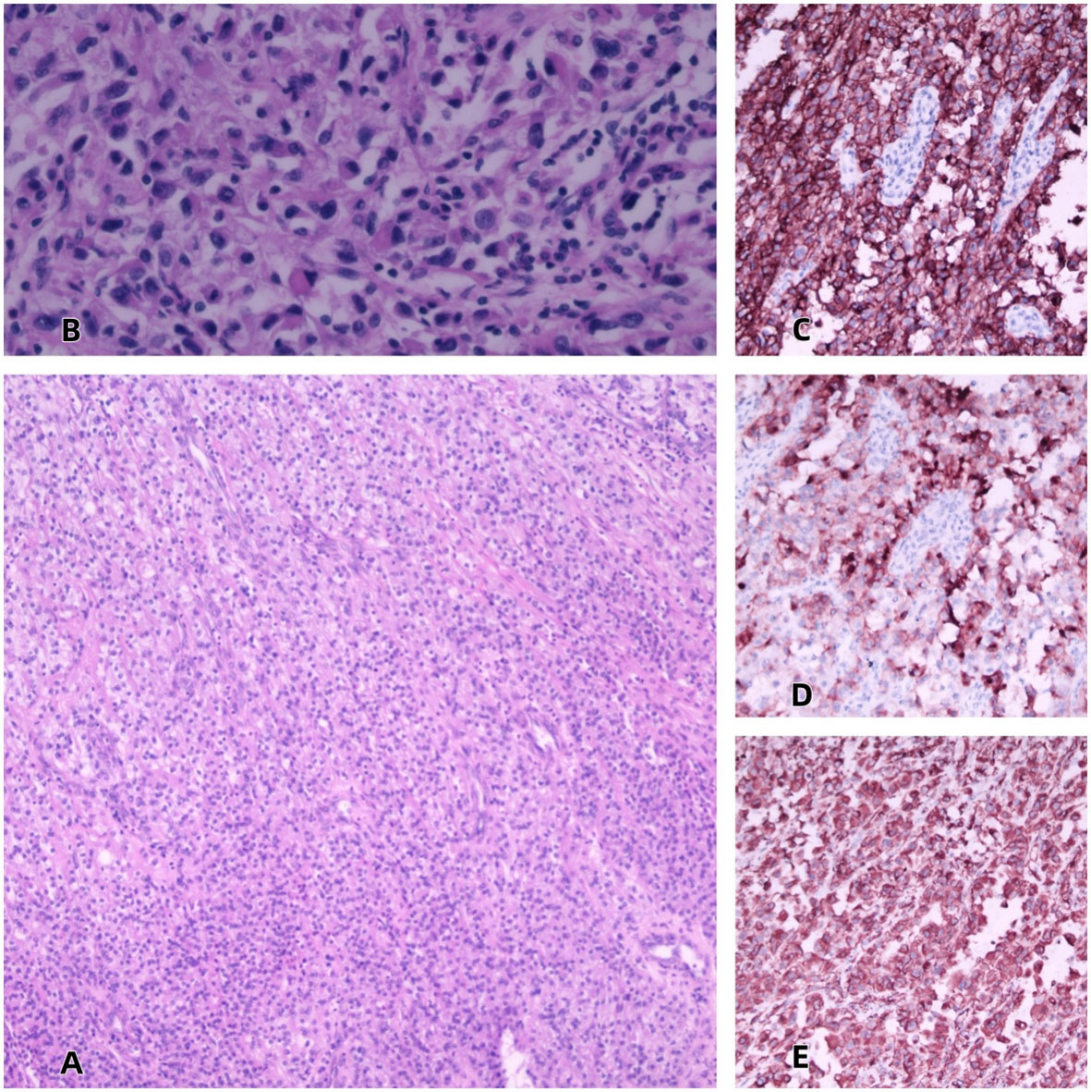
**(A)** HE 10× **(B)** HE 40× **(C)** CAIX 20× **(D)** CD10 20× **(E)** Vimentin 20×.

Seven days after the operation, the pathology department of our hospital returned the pathological examination results: (Left kidney and tumor) combined with immunohistochemistry, renal clear cell carcinoma with necrosis, volume of 12*10*8cm, World Health Organization/International Society of Urological Pathology classification(WHO/ISUP)grade III mostly, local grade IV, involving renal pelvis, renal capsule, perirenal fat, tumor thrombus in renal portal vein, no definite nerve metastasis. The ureteral stump was positive, and cancer tissue was found in the adrenal gland, and no cancer was found in the retroperitoneal lymph nodes (0/2). (Bladder tumor) Carcinoma component was found in fibrous tissue, which was considered to be homologous to renal tumor. histopathologic stage: pT3N0M1.Immunohistochemical staining:CD10(+),Vimentin(+),CAIX(+),CK7(-),CD117(Focal+),E-Cadherin(-),RCC(Weakly+),GATA3(Focal+),CK5/6(-),P40(-),TFE3(Weakly+),CK20(-),ki-67(Index 50%). (**Figure 4**).

We planned to give the patient a target-immune combination regimen of axitinib 5mg bid and toripalimab 240mg q3w for subsequent therapy after the surgical incision had completely healed three weeks after surgery.

The patient began the first postoperative follow-up on February 18, 2026, with the main purpose of initiating the relevant targeted and immune combination therapy as discussed above. Subsequently, the patient regularly received an injection of toripalimab every three weeks and took axitinib orally twice a day at a dose of 5mg until April 1, 2026, when they returned to the hospital for another round of immunotherapy. At that time, plain CT scans of the abdomen and chest were performed. The abdominal CT scan showed that there was no abnormal mass in the original surgical area after left kidney surgery, and the lymph nodes in the retroperitoneum were slightly enlarged, with no significant change compared to before. The results of the lung CT scan were more encouraging. Although multiple metastases still existed, all the metastatic lesions had shrunk, with the largest one reducing from 1.2cm to about 0.9cm. Moreover, the patient no longer experienced any discomfort such as fever or hematuria, indicating the effectiveness of the cytoreductive surgery.

## Discussion

For this case, the key difficulty lies in differentiating it from urothelial carcinoma, as the latter more readily exhibits the characteristic of invasion along the urinary tract. The patient’s CAIX, CD10, and Vimentin are all positive. Current research has demonstrated that Vimentin can serve as an effective tumor marker for renal cell carcinoma, with its expression significantly elevated in renal tumor cells. It forms a complex with the p53 gene in the cytoplasm of renal tumor cells, thereby inhibiting the pro-apoptotic function of p53 (7, 8). CD10 has also been proven to be a tumor marker for clear cells, highly expressed in clear cell renal cell carcinoma (9, 10). CAIX, on the other hand, can effectively distinguish whether the tumor cells are of renal tubular epithelial cell origin, thereby differentiating renal cell carcinoma from urothelial carcinoma (11).

Unlike localized RCC, RCC involving the bladder often presents as gross hematuria (12), while localized RCC usually has no obvious specific clinical manifestations and is often incidentally discovered during physical examination.

In addition to local spread to perirenal fat (pT3) or perirenal fascia (pT4), RCC can also metastasize along the venous system, but the prognosis is often poor. Some data also suggest that invasion of the collecting system by renal cell carcinoma is an independent factor affecting prognosis (13).

The mechanism of ureteral involvement in renal cell carcinoma remains controversial. Currently, three theories have been proposed: 1. Renal cancer involving the ureter; tumor cells grow directly along the urinary tract lumen and invade the bladder. This is because the pressure in the renal collecting system increases after the formation of venous tumor thrombus, and the tumor cells are affected by venous pressure and enter the renal collecting system. Some scholars have also found renal cancer cells in the urine of some patients with renal cancer, and based on this observation, the dissemination of tumor cells through the urinary tract may be a reasonable explanation for the forward spread of renal tumors to the ureter and bladder; 2. Renal cell carcinoma tumor cells flow retrograde to the bladder through the venous tumor thrombus. Researchers supporting this theory point out that the proportion of pelvic metastasis (including metastasis to the bladder, ureter, testis, and vagina) in left renal cell carcinoma is higher, and they attribute this to the richer pelvic venous communication on the left side. Some data even suggest that part of the left renal vein communicates with the left lumbar vein, which in turn communicates with the vertebral venous plexus and intracranial veins. Therefore, tumor cells from left renal tumors can undergo retrograde intracranial metastasis; 3. Some data suggest that lymphatic metastasis may be involved (6, 12, 14). Here, the author only found 4 cases that described ureteral tumor thrombus and whether they were accompanied by venous tumor thrombus. Among them, only 1 case was accompanied by venous tumor thrombus, and 3 cases were not (6, 15, 16). Due to the extremely small amount of data, there is no statistical significance. Here, the author suggests that clinicians should pay attention to this aspect and keep good statistical records to determine whether the formation of ureteral tumor thrombus is related to the formation of venous tumor thrombus.

Clinically, renal cell carcinoma with ureteral tumor thrombus should be differentiated from urothelial carcinoma. More than 90% of malignant tumors of the renal pelvis and ureter are renal pelvis cancer, while the pathological types of renal cell carcinoma with ureteral tumor thrombus should be paid attention to. Some data have been statistically analyzed. Jeff John et al.’s article described 4 cases, of which 3 were clear cell renal cell carcinoma (RCCC), and one was clear cell renal cell carcinoma (RCCC) combined with papillary renal cell carcinoma (pRCC) (17). Yu Tian et al.’s article described 22 cases, 15 of which were RCCC, and a few were papillary renal cell carcinoma (pRCC), chromophobe renal cell carcinoma (cRCC), and other types (18). Clear cell carcinoma accounts for about 70%, which is consistent with the fact that clear cell carcinoma accounts for 70% to 85% of all renal malignant tumors (19). However, due to the small sample size, no conclusion can be drawn on whether the formation of ureteral tumor thrombus is directly related to the pathological type of renal malignant tumors. However, from the small sample, it seems that the formation of ureteral tumor thrombus is not related to the pathological type. Clear cell renal cell carcinoma (RCCC) has a typical golden-yellow appearance; grossly, it is often accompanied by necrosis, hemorrhage, and cystic changes. The tumor margin is often pushed, and the cut surface can be variegated, accompanied by glassy fibrosis, necrosis, and hemorrhage. Some authors believe that the main route of spread of clear cell renal cell carcinoma (RCCC) is through fat infiltration of the renal sinus (20), which seems to confirm the fact that clear cell renal cell carcinoma rarely forms ureteral tumor thrombus.

For solitary stage I, II, and some stage III RCCC, surgical treatment remains the preferred option; solitary, easily resectable, and slowly progressing metastases can also be resected surgically. For advanced RCCC that cannot be treated by surgery, tyrosine kinase inhibitors (TKIs) targeted therapy combined with immunotherapy is often adopted (19). Here, the author retrieved 26 cases, including 11 cases of unilateral radical nephrectomy and 15 cases of unilateral nephroureterectomy (6, 14, 16–18). However, due to the small sample size, the author was unable to determine which surgical approach had a better prognosis or to assess the efficacy of postoperative combined targeted and immunotherapy. In summary, in terms of treatment, clinicians tend to use surgical methods and treatment plans for middle and advanced stage renal cancer.

## Conclusions

Cases of renal malignant tumors combined with ureteral tumor thrombus even reaching the bladder are relatively rare in clinical practice. Its diagnosis can rely on Computed Tomography (CT) and Magnetic Resonance Imaging (MRI). However, its clinical significance and standardized treatment are still under exploration. Although the specific surgical procedure mostly depends on the experience of the clinician, but some cases still prove that surgery is an effective treatment.

## Data Availability

The original contributions presented in the study are included in the article/**Supplementary Material**. Further inquiries can be directed to the corresponding authors.
