# Supplementary figures and images for: Renal clear cell carcinoma with co-existing tumor thrombosis of renal vein and ureter: a case report and review of the literature
Source: Front Oncol. 2026 May 13;16:1844930. doi: 10.3389/fonc.2026.1844930 (PMC13212044; doi:10.3389/fonc.2026.1844930)

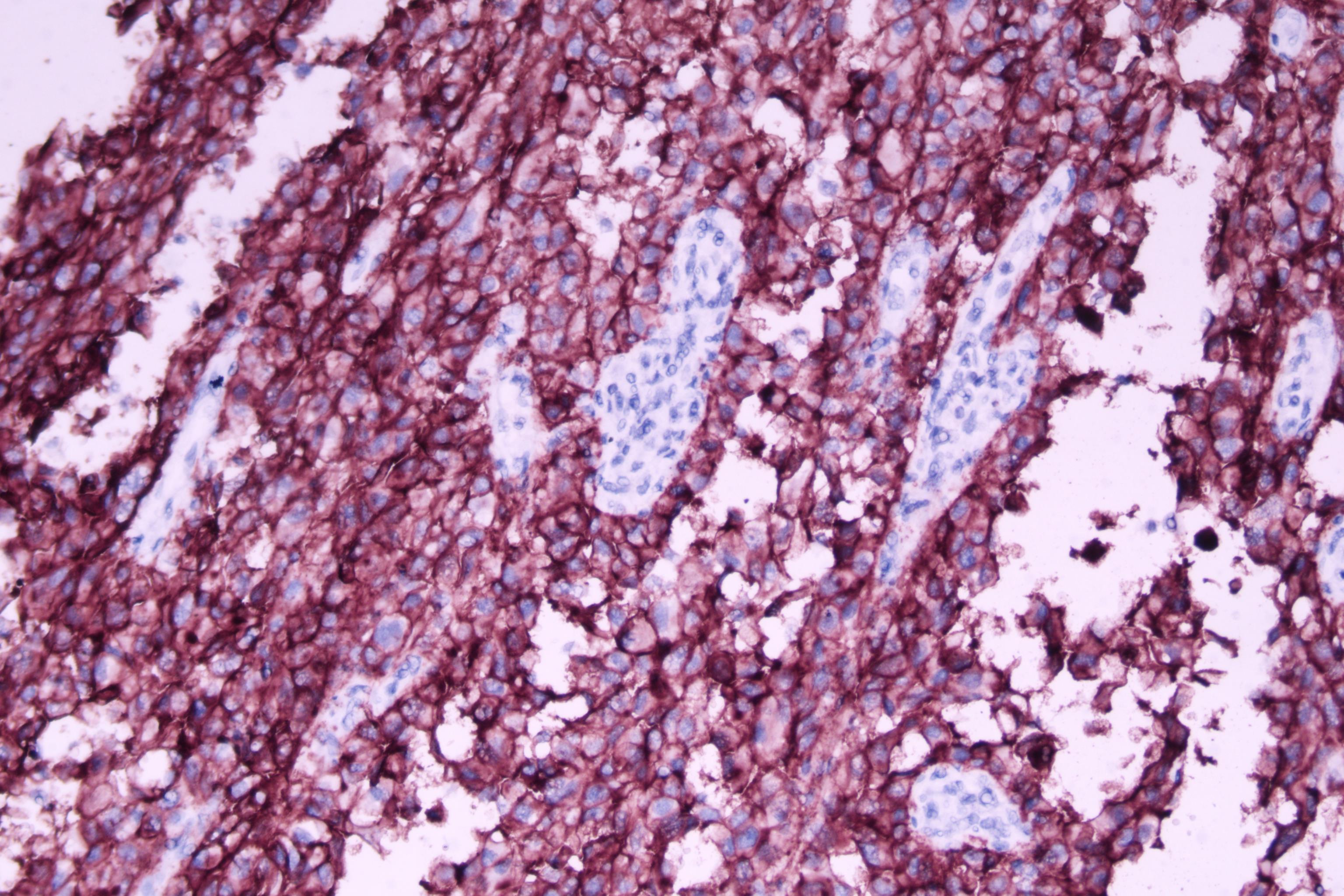

Supplement: Supplementary file 1 [file DataSheet1.zip › pathology figure/S2601812-CAIX 20X-20260330-134004-754.jpg]

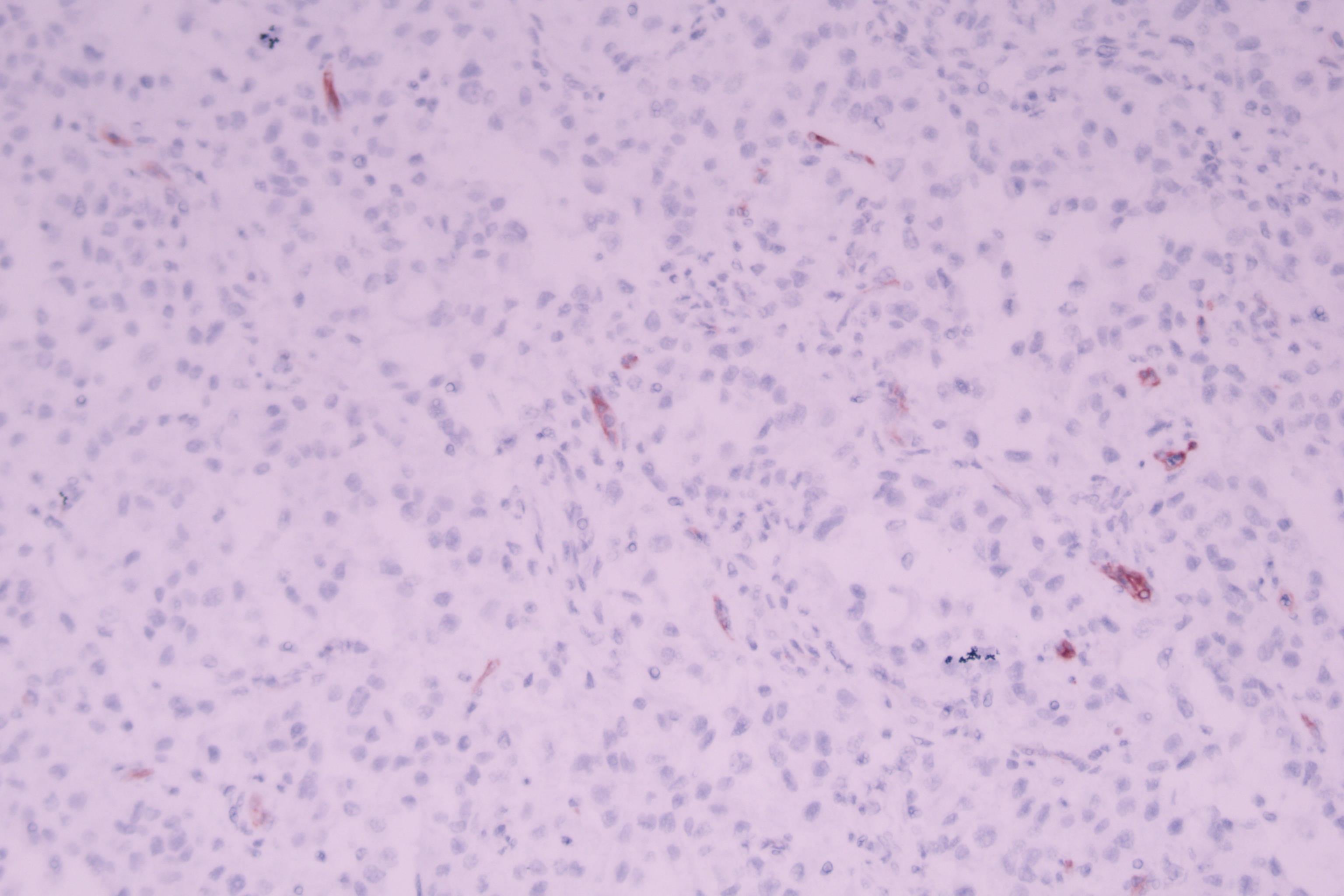

Supplement: Supplementary file 1 [file DataSheet1.zip › pathology figure/S2601812-CD117 20X-20260330-134004-749.jpg]

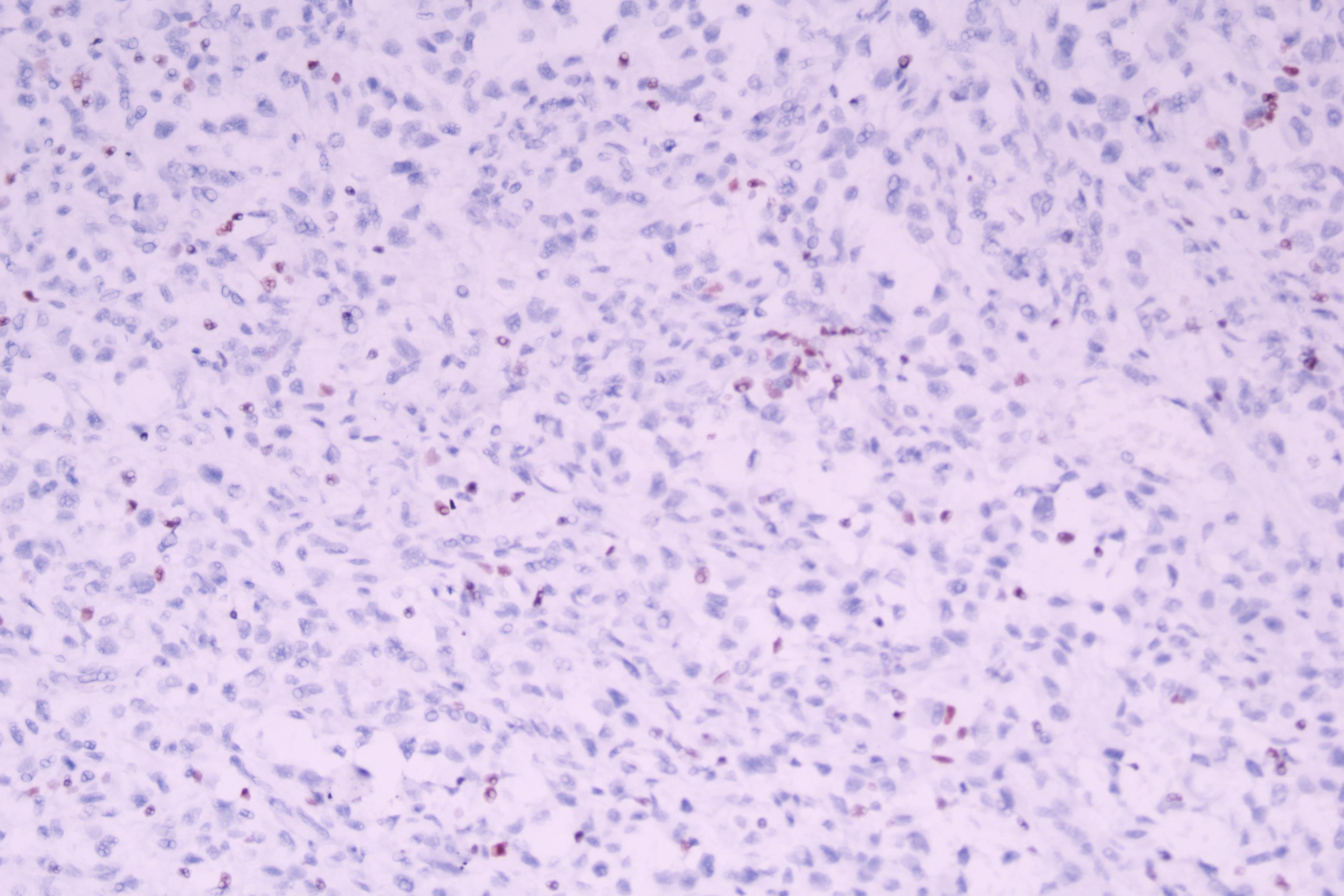

Supplement: Supplementary file 1 [file DataSheet1.zip › pathology figure/S2601812-GATA3 20X-20260330-134004-752.jpg]

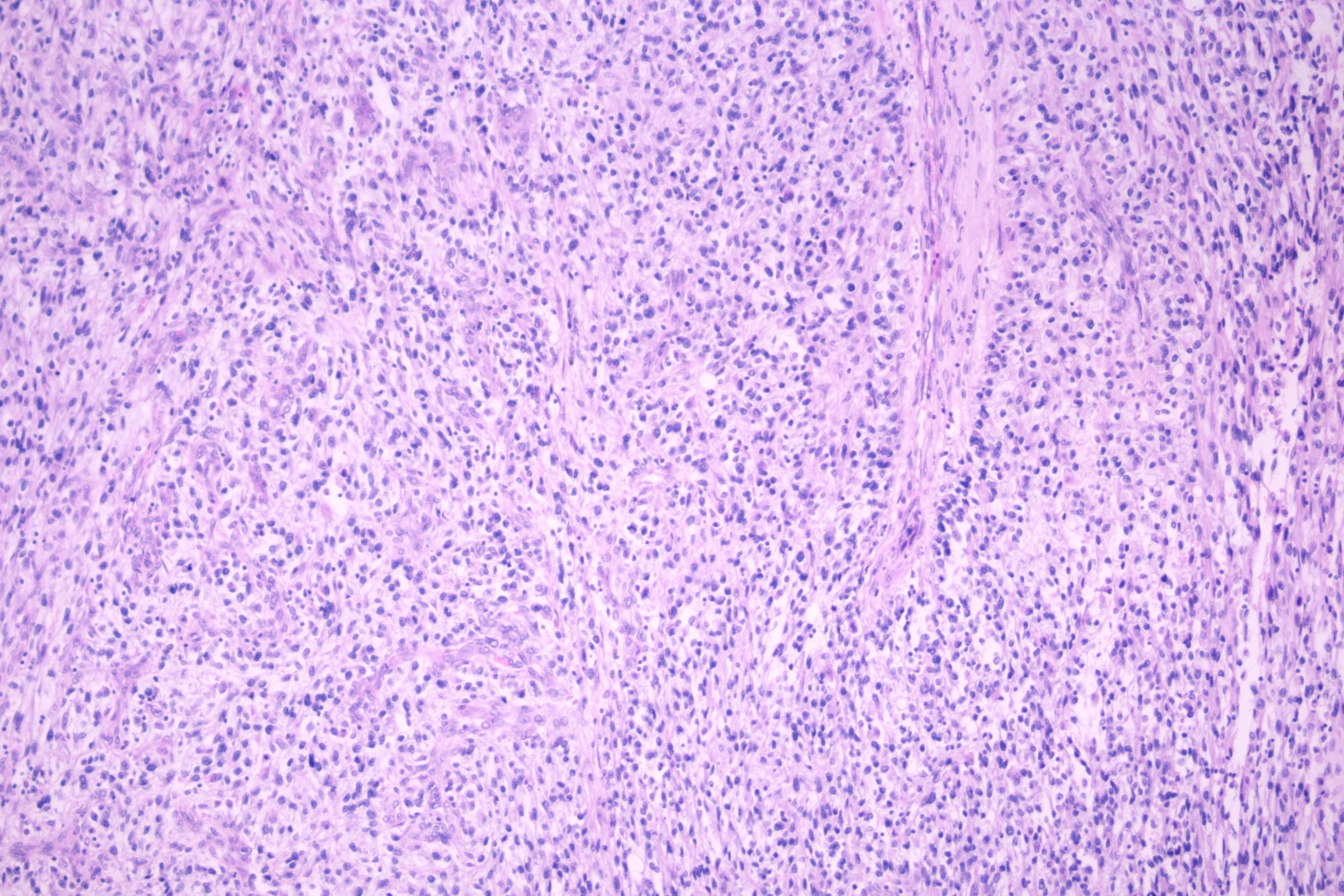

Supplement: Supplementary file 1 [file DataSheet1.zip › pathology figure/S2601812-HE 10X-20260330-134004-766.jpg]

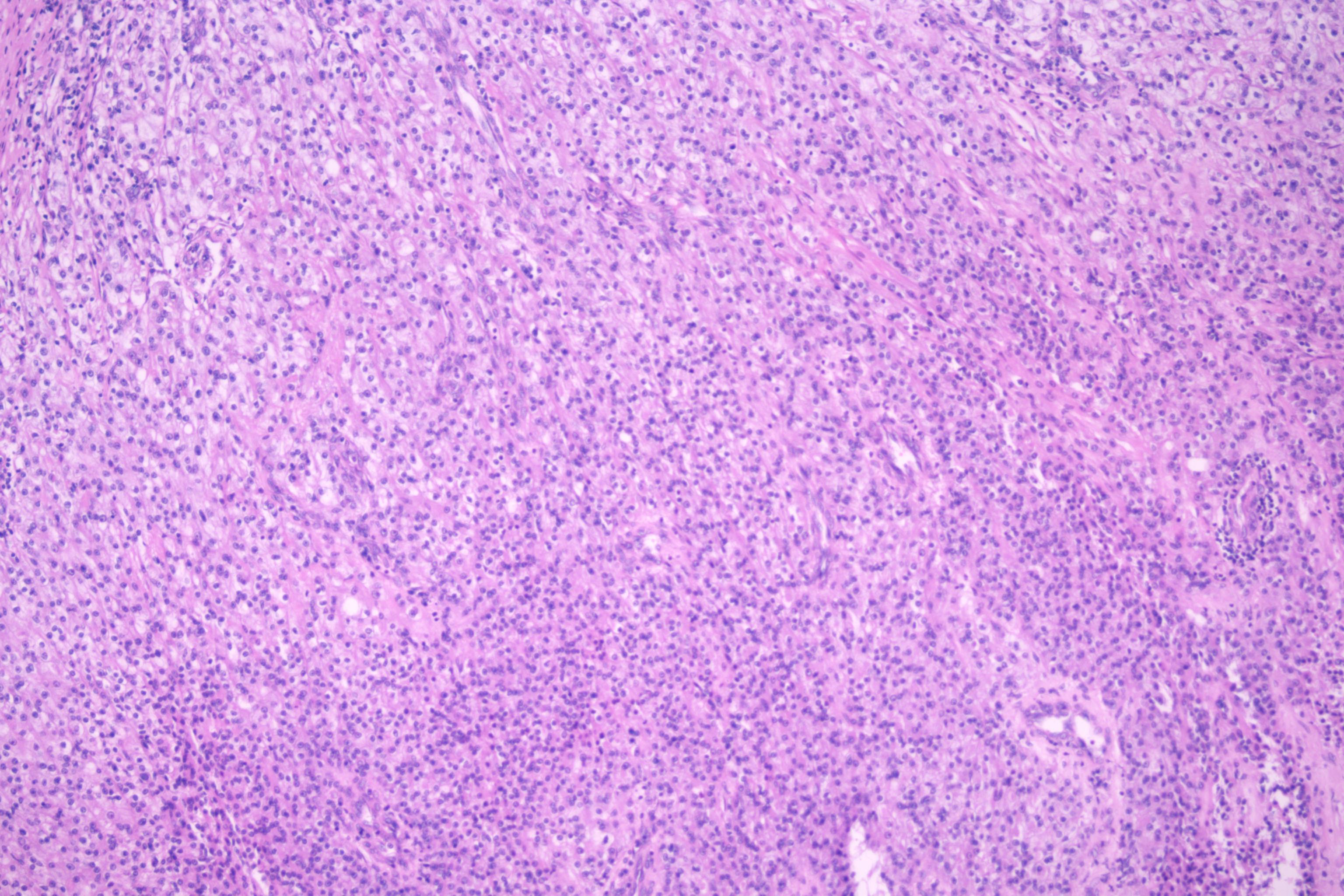

Supplement: Supplementary file 1 [file DataSheet1.zip › pathology figure/S2601812-HE 10X-20260330-134004-767.jpg]

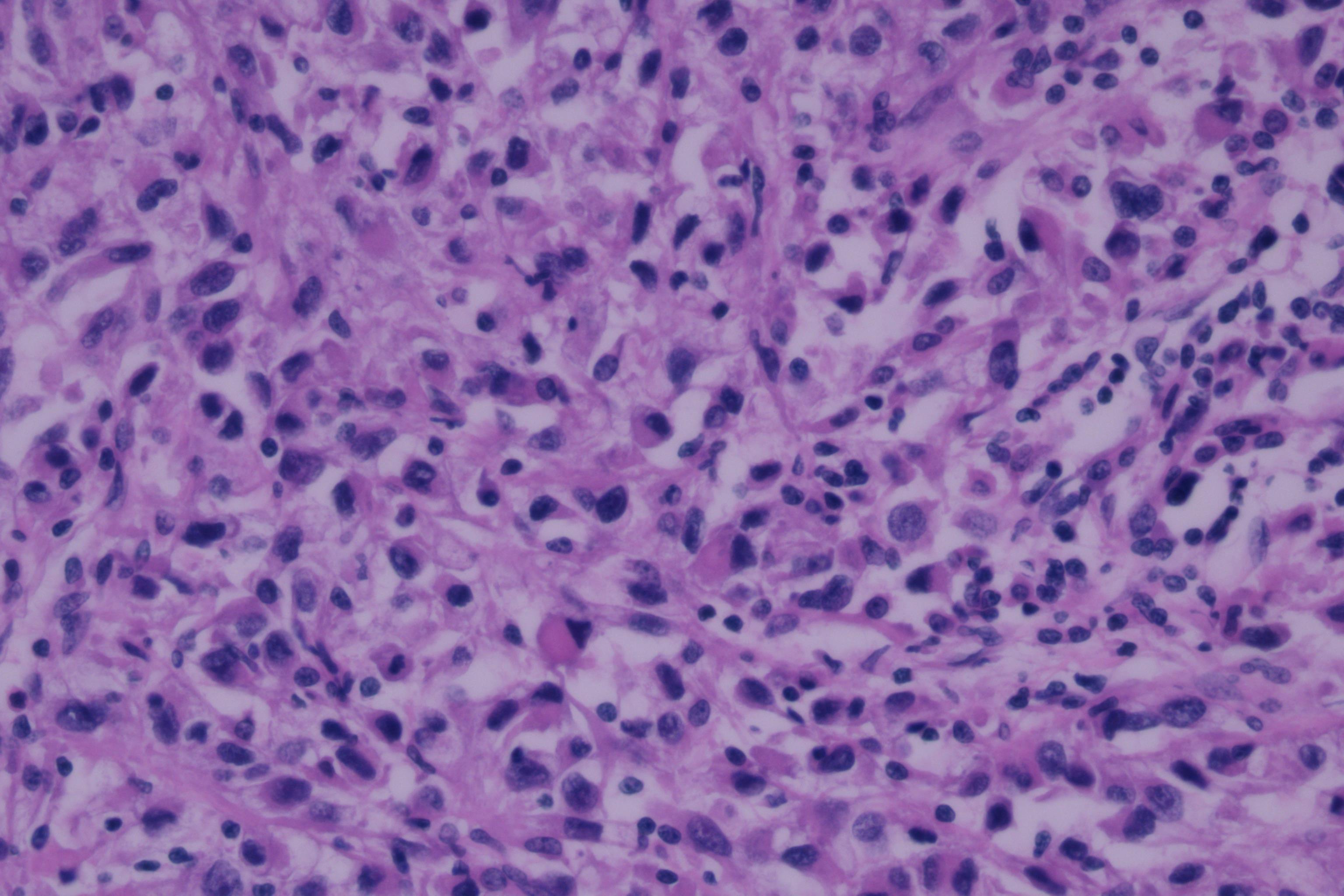

Supplement: Supplementary file 1 [file DataSheet1.zip › pathology figure/S2601812-HE 40X-20260330-134004-758.jpg]

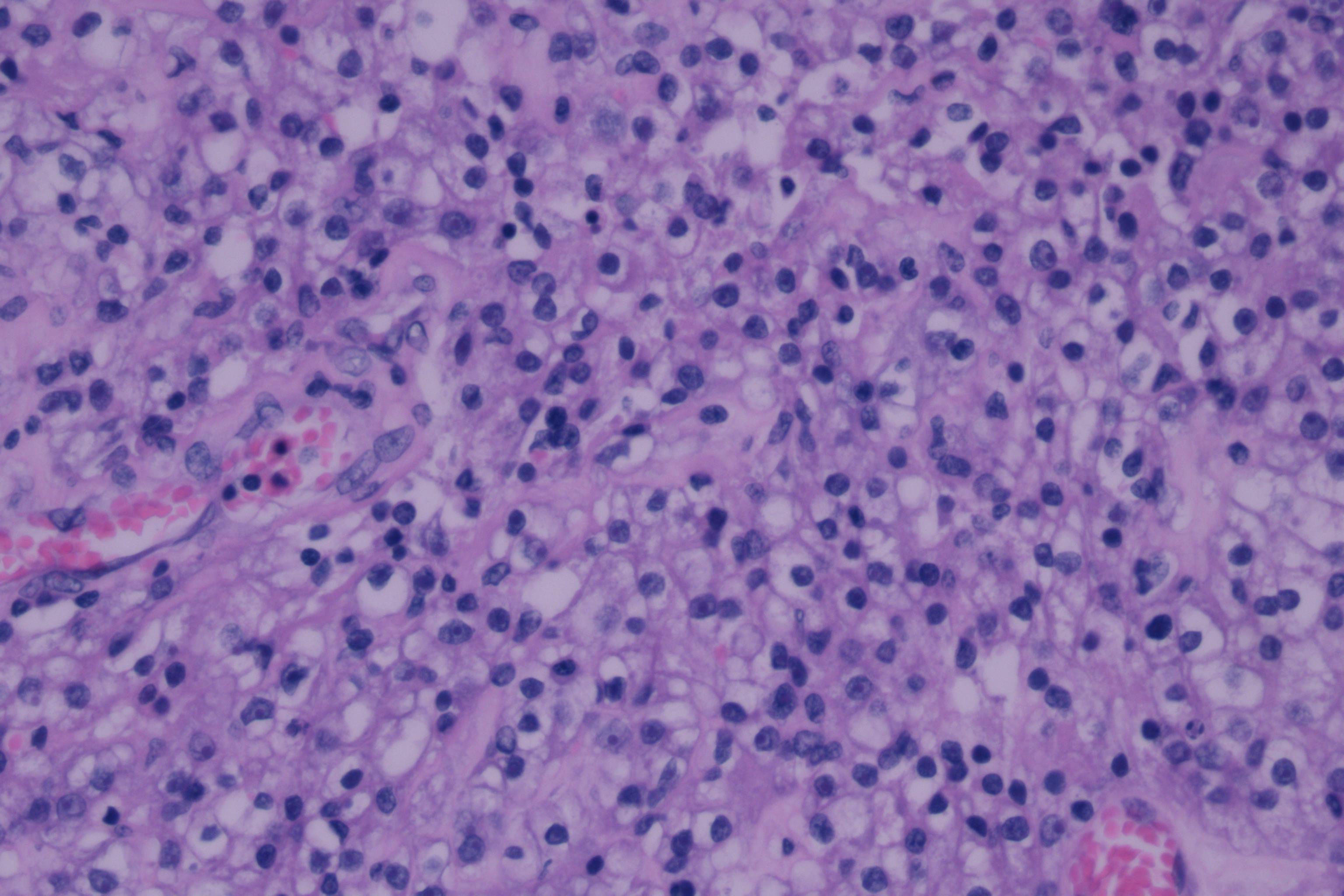

Supplement: Supplementary file 1 [file DataSheet1.zip › pathology figure/S2601812-HE 40X-20260330-134004-759.jpg]

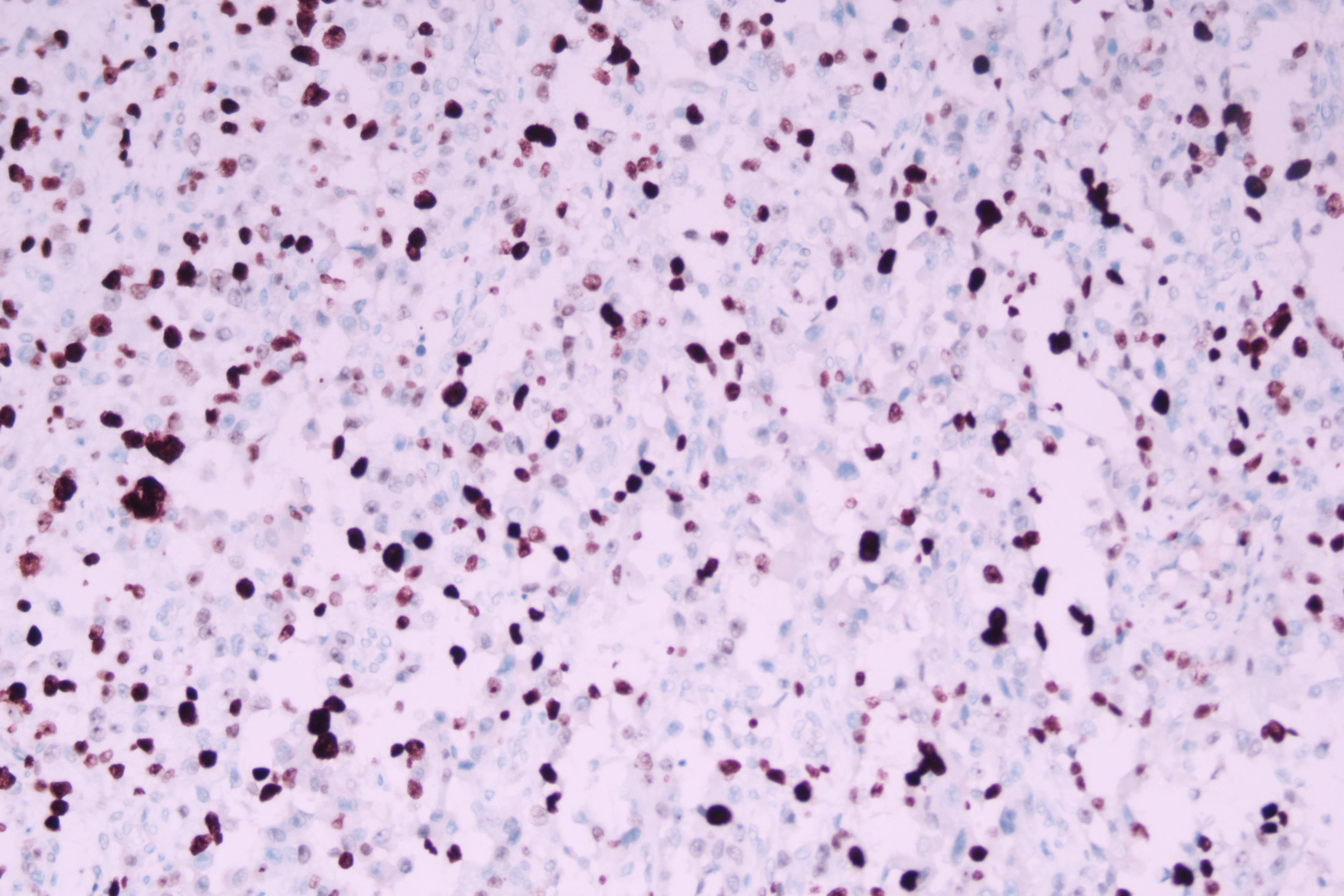

Supplement: Supplementary file 1 [file DataSheet1.zip › pathology figure/S2601812-Ki-67 20X-20260330-134004-750.jpg]

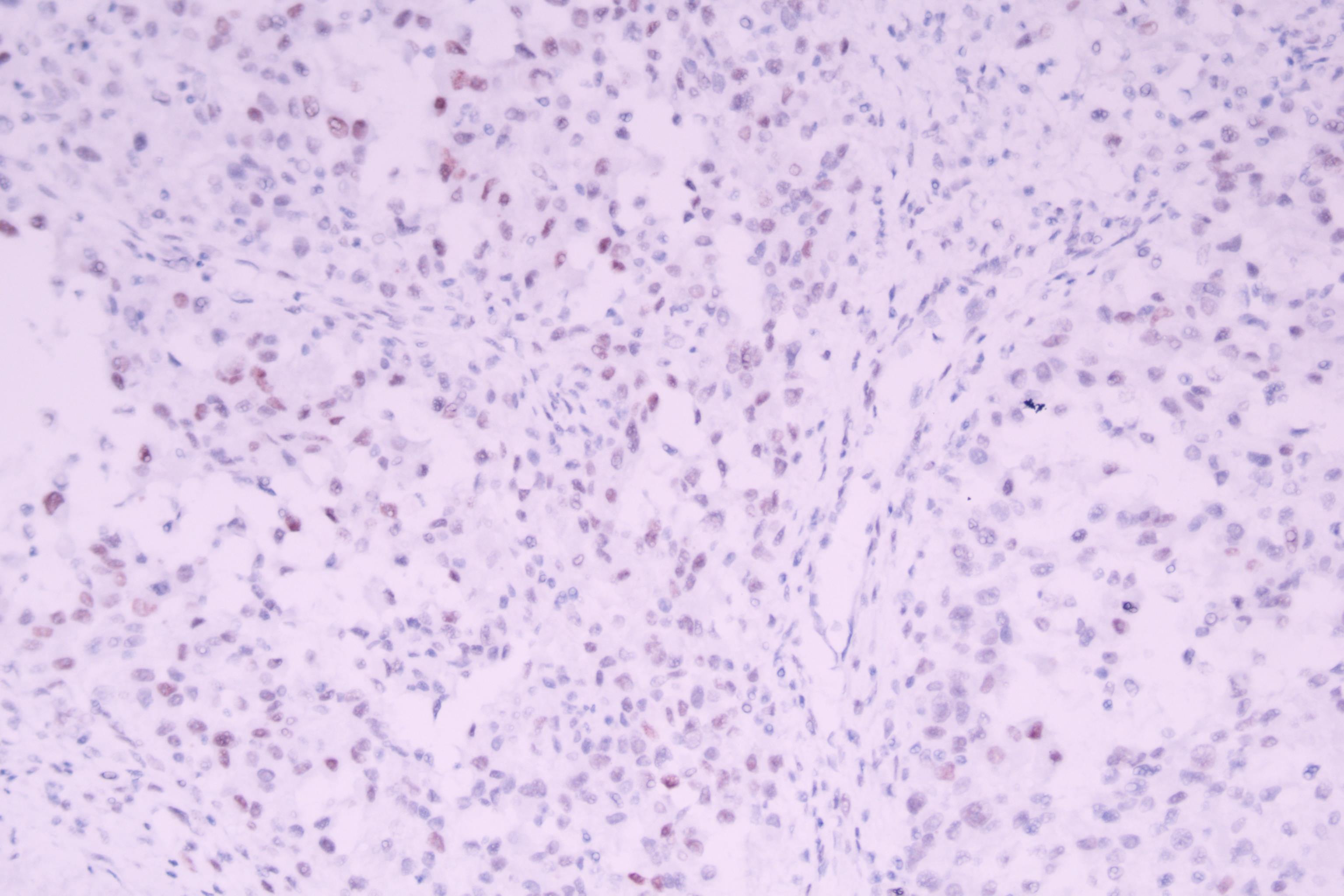

Supplement: Supplementary file 1 [file DataSheet1.zip › pathology figure/S2601812-TFE-3 20X-20260330-134004-751.jpg]

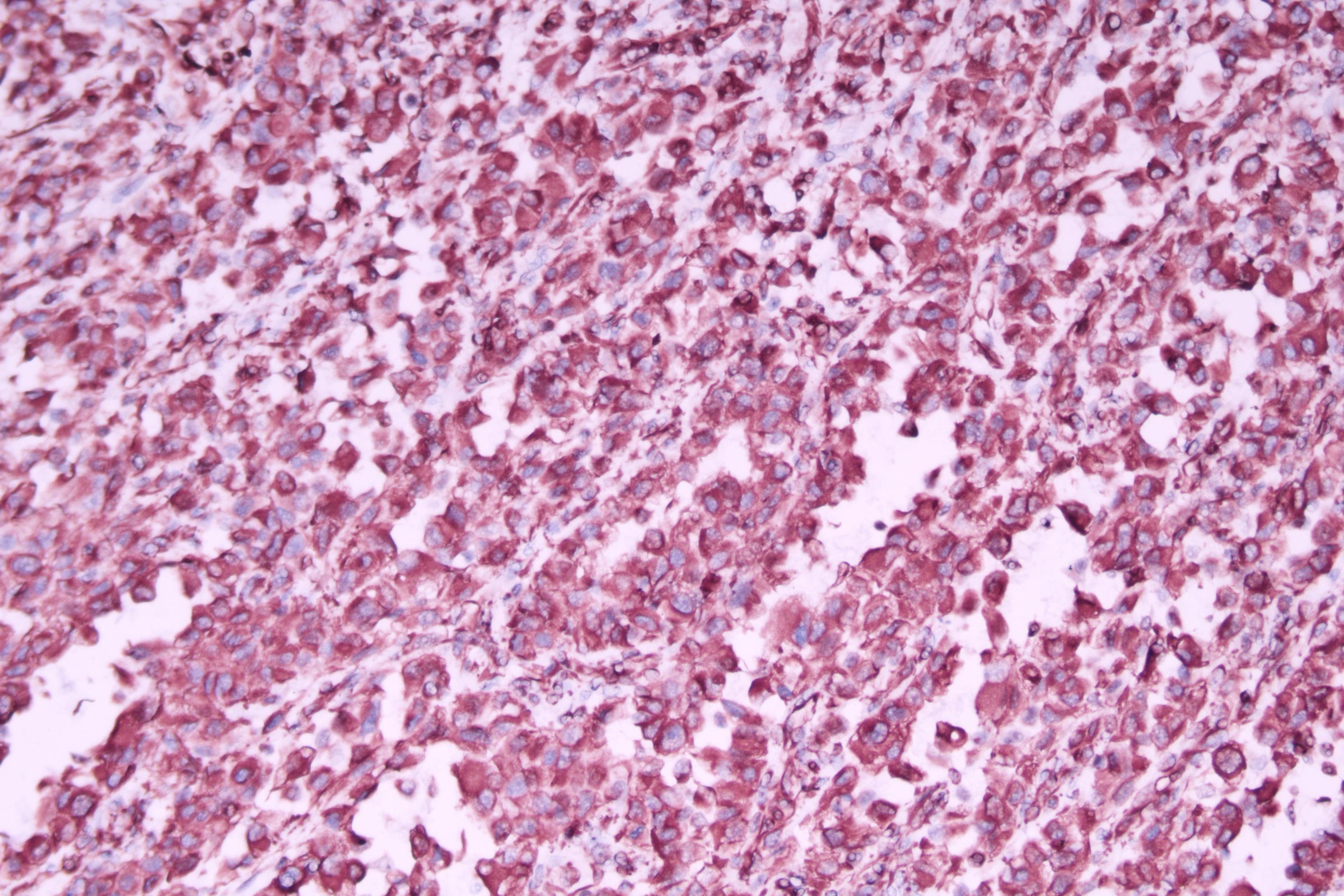

Supplement: Supplementary file 1 [file DataSheet1.zip › pathology figure/S2601812-Vimentin 20X-20260330-134004-755.jpg]
